# Interstitial Lung Disease Secondary to Sjogren’s Syndrome and Antisynthetase Syndrome: Converging Disease Trajectories

**DOI:** 10.3390/medicina61112044

**Published:** 2025-11-15

**Authors:** Giuseppe Muscato, Giulia Morina, Evelina Fagone, Mary Fruciano, Elisa Gili, Serafina Martella, Stefano Palmucci, Domenico Sambataro, Carlo Vancheri, Gianluca Sambataro

**Affiliations:** 1 Regional Referral Centre for Rare Lung Diseases, University Hospital Policlinico “G. Rodolico-San Marco”, Department of Clinical and Experimental Medicine, University of Catania, 95124 Catania, Italy; giuseppe.muscato@phd.unict.it (G.M.); eva.fag@virgilio.it (E.F.); maryfruciano@yahoo.it (M.F.); seramartella@gmail.com (S.M.);; 2 Unità Operativa Semplice Dipartimentale di Imaging Polmonare e Tecniche Radiologiche Avanzate (UOSD I.P.T.R.A.), University Hospital Policlinico “G. Rodolico-San Marco”, Department of Medical Surgical Sciences and Advanced Technologies “GF Ingrassia”, University of Catania, 95124 Catania, Italy; 3 Artroreuma S.R.L., Outpatient Clinic Associated with the Regional Health System, 95030 Mascalucia, Italy; 4 Department of Medicine and Surgery, University of Enna “Kore”, 94100 Enna, Italy

**Keywords:** Sjogren Syndrome, Antisynthetase Syndrome, interstitial lung diseases, CTD-ILD, anti-Ro52kD antibody

## Abstract

*Background and Objectives*: Interstitial lung disease is one of the main causes of mortality in Sjögren’s Syndrome (SjS) and Anti-Synthetase Syndrome (ASyS). The objective of the study is to compare clinical, serological and radiological features of these conditions, as well as their prognosis. *Materials and Methods*: we retrospectively enrolled 34 SjS-ILD and 33 ASyS-ILD patients. The two cohorts were jointly followed by rheumatologists and pulmonologists for at least two years. *Results*: From a clinical point of view, ASyS-ILD patients showed a greater prevalence of myositis (18.2% vs. 2.3%, *p* = 0.04), whereas more SjS patients had sicca syndrome (85.3% vs. 9.1%, *p* < 0.001). No other clinical differences were noted. From a serological point of view, ASyS-ILD patients had a greater proportion of antinuclear antibody positivity with a cytoplasmic pattern (24.9% vs. 2.9%, *p* = 0.005) and positivity for Anti-Synthetase Antibodies (ASA), which were not found in the SjS cohort. SjS-ILD patients were mainly positive for anti-Ro52kD (61.8% vs. 27.3%, *p* = 0.002). No significant differences were noted in radiological pattern of ILD, functional values, disease progression and prognosis. *Conclusions*: SjS-ILD and ASyS patients show several common features. It could be hypothesized that some patients classified as SjS-ILD could have undetectable or unknown ASA, and the upcoming ASyS classification criteria may be useful in highlighting these patients for deeper research.

## 1. Introduction

Interstitial lung disease (ILD) is a condition characterized by the deposition of extracellular matrix and/or inflammatory cells in the lung interstitium, potentially leading to respiratory failure and death. Autoimmune conditions represent about 30% of total ILD patients, and in those patients, lung involvement is one of the leading causes of mortality [1,2]. The correct classification of ILD as secondary to autoimmune conditions has great prognostic value: autoimmune ILDs generally have a better prognosis than Idiopathic Pulmonary Fibrosis (IPF), and the treatment could benefit from immunosuppressants even in the presence of a clear fibrotic ILD pattern [3]. Unfortunately, diagnosis is not always simple: despite ILD showing a prevalent pattern for each condition, for example, usual interstitial pneumonia (UIP) in Rheumatoid Arthritis (RA) and fibrotic Nonspecific Interstitial Pneumonia (NSIP) in Systemic Sclerosis (SSc), all the radiological patterns are possible in each condition [2]. Moreover, at least in respiratory settings in which lung involvement is prevalent, other rheumatological signs can be nuanced, and a significant proportion of patients are seronegative for specific autoantibodies [4]. Hence, at present, the presence of ILD is not deemed sufficiently specific for the classification of a high number of systemic autoimmune diseases, being included only in the classification criteria for SSc, microscopic polyangiitis and the upcoming criteria for Anti-Synthetase Syndrome (ASyS) [5,6,7,8].

The recognition of Sjögren’s Syndrome (SjS) and ASyS in ILD patients is important to drive appropriate treatment but is not always straightforward because the presentation of the two conditions tends to be incomplete when lung involvement is present.

SjS is a potentially systemic Connective Tissue Disease (CTD) mainly characterized by an autoimmune response against the exocrine glands, clinically associated with xerophthalmia and xerostomia. The proportion of SjS patients with ILD is about 20%, one of the lowest proportions compared with other CTDs [9]. As already reported in other CTDs, all the radiological patterns on High-Resolution Computer Tomography (HRCT) can be associated with this condition. Lymphocytic Interstitial Pneumonia (LIP) is a rare although highly specific pattern, but the most common is NSIP, potentially with a fibrosing feature, given that about half of SjS ILD patients can show a progressive-fibrosing phenotype (PFP), increasing mortality by about 3 times [9,10]. From a clinical point of view, SjS-ILD patients generally report no or only mild sicca symptoms, less involved histological samples from minor salivary gland biopsy, and a lower proportion of seropositivity, mainly for anti-Ro52kD rather than anti-Ro60kD [9]. This clinical picture is mainly described in SjS-ILD patients in which ILD precedes the development of a definite SjS: in those patients, the prevalent ILD pattern is UIP, commonly seronegative and without significant xerophthalmia or xerostomia; therefore, these patients are commonly classified as IPF [11].

ASyS is an idiopathic inflammatory myopathy (IIM) characterized by the classic triad including inflammatory arthritis, myositis and ILD, associated with seropositivity for Anti-Synthetase Antibodies (ASA). In this triad, the most common sign is ILD, reported in about 80% of patients [12]. ILD is also the first cause of mortality in ASyS, mainly in patients with non-anti-Jo1-ASA [13]. As reported in SjS, also in this case is the prevalent pattern is NSIP, potentially overlapping with Organizing Pneumonia (OP); however, a PFP is noted in about 40% of patients [12,14]. Beyond ASAs, as the specific serological marker of the condition, anti-Ro52kD can be present in about 50% of ASyS patients and associated with the presence of ILD [15]. The recognition of an underlying ASyS in ILD patients is not always straightforward, given that up to 40% of ASyS patients only show ILD at the time of diagnosis, developing other signs of the classic triad during follow-up, whereas up to 10% of patients might show only ILD during their whole clinical history [16]. Those incomplete clinical pictures are potentially classified as Interstitial Pneumonia with Autoimmune Features (IPAF), a research classification including ILD patients with clinical and/or serological autoimmune signs not sufficient to reach the classification criteria for autoimmune conditions [17].

Therefore, studies report similar radiological features and prognoses in ILD associated with SjS and ASyS, as well as similar clinical presentations in which ILD can be the first or even the sole clinical sign of the condition. In view of this, it is not surprising that about 40% of IPAF patients in prospective cohorts will develop SjS or ASyS during their clinical history [18].

The aim of the study is to compare ILD patients with SjS and ASyS, looking for possible differences and similarities in ILD presentation, prognosis and risk factors.

## 2. Materials and Methods

We conducted a retrospective, single-center study at the Regional Referral Center for Rare Lung Disease, University of Catania. All patients or their families (in case of the patient’s death) provided informed consent for the anonymous management of retrospective data. The study was conducted according to the Declaration of Helsinki, updated to the latest version and approved by the local ethical committee “Catania 1” (CT1, 12/07/22–1565).

### 2.1. Patient Enrollment

For enrollment we used our database, considering all consecutive ILD patients with a diagnosis of SjS or ASyS made from January 2017 to August 2023, followed by 2 years of follow-up. Inclusion criteria for enrollment were age ≥18 years, diagnosis of SjS or ASyS, presence of ILD proven by HRCT and presence of written informed consent to the management of retrospective data. Exclusion criteria were the absence of the inclusion criteria, presence of overlapping autoimmune diseases and missing data >10%. The classification of SjS or ASyS was made in accordance with the available classification criteria. For SjS we used the validated classification criteria, whereas for ASyS, for which no validated criteria are currently available, we used the Connors criteria or the contemporary presence of the complete ASyS classic triad together with ANA positivity with a cytoplasmic pattern [19,20]. The diagnosis of autoimmune diseases was made by rheumatologists working together with pulmonologists on the same team, or on the weekly multidisciplinary team, including rheumatologists, radiologists and pulmonologists. In these regular meetings, all doubtful cases were discussed to reach a shared decision from a diagnostic and therapeutic point of view. In any case, all the patients were clinically evaluated by both rheumatologists and pulmonologists within three months of the first visit or checkup; therefore, the clinical characteristics registered in these patients were considered contemporary if reported in this time range. We considered patients with a minimum follow-up of 24 months to evaluate ILD progression over time. Patients that developed other systemic autoimmune conditions during the follow-up were excluded as well. Deaths were ascertained from clinical records, and causes were classified as respiratory or non-respiratory.

### 2.2. Serological Assessment

All ILD patients were evaluated with the same serological approach including complete blood count, Erythro-Sedimentation Rate, C Reactive Protein, ferritin, myoglobin, Creatine Phosphokinase, Lactic Dehydrogenase, creatinine, transaminases, complement fractions, serum protein electrophoresis Antinuclear Antibodies (ANA), Anti Neutrophil Cytoplasmic Antibodies, anti DsDNA, anti-citrullinated protein antibodies, rheumatoid factor, Extractable Nuclear Antigens (ENA) profiles including anti-Ro52kD, anti-Ro60kD, anti-La, anti-RNP, anti-Sm, anti-Scl70 and anti-centromere antibodies. ANA was studied with indirect immunofluorescence with seropositivity considered for a minimum titer of 1:80, whereas ENA profile was conducted with commercial immunoblotting kits. The study for myositis-specific and myositis-associated antibodies (MSA and MAA, respectively), included anti-PL7, anti-PL12, anti-Pm/scl, anti-MDA5, anti-SRP, anti-Mi2, anti-Tif1γ, anti-Ku, anti-EJ and anti-OJ. According to our internal guidelines, it was performed on patients with one or more of the following features: proximal weakness, significant myalgia, increased muscle enzymes, presence of NSIP with or without OP, ANA positivity with a cytoplasmic pattern, anti-Ro52kD positivity, Nailfold Videocapillaroscopy (NVC) positivity and/or presence of bushy capillaries, including other possible indications of concurrent myositis such as myopathic pattern in Electromyography or presence of suspected myositis on Magnetic Resonance Imaging [2,21]. Therefore, all the patients enrolled in this study were tested for these autoantibodies. MSA and MAA were evaluated also using commercial immunoblotting kits (Euroline inflammatory Myopathies 16 Ag, Euroimmun, Lubeck, Germany). The general serological assessment was repeated periodically to evaluate response and tolerability of immunosuppressive treatment, whereas the autoimmune profile was repeated annually.

### 2.3. Instrumental Assessment

NVC was performed on all patients, with or without Raynaud’s Phenomenon (RP), considering that NVC positivity in patients with IIM can be present even in the absence of RP [22]. It was defined “positive” in the presence of giant capillaries or avascular areas and classified according to the specific guidelines; however, bushy capillaries were also reported. The exam was primarily used to exclude underlying scleroderma spectrum disorder (alone or in overlap) but also as an ancillary exam, potentially being able to suggest the presence of IIM also through the presence of bushy capillaries [23,24].

Tests for the evaluation of exocrine gland function included in the SjS classification criteria (e.g., Schirmer Test, salivary gland biopsy) were performed during the rheumatologic assessment in ILD patients with sicca syndrome or seropositivity for anti-Ro. Occasionally, Electromyography or other instrumental exams were performed to evaluate the presence of myositis.

Pulmonary Function Tests and 6 Min walking tests were conducted on all patients at the first assessment and every 3–6 months during follow-up during the respiratory assessment, following the specific guidelines [25,26,27].

HRCT was performed on all patients to confirm the diagnosis of ILD. The radiological pattern was classified according to the current guidelines by an expert thoracic radiologist that visually analyzed all the exams. HRCT was repeated annually if clinically indicated and, together with the clinical symptoms and functional assessment, was used to classify patients as having PFP according to the definition of Progressive Pulmonary Fibrosis (PPF) proposed in the 2022 clinical practice guideline published by ATS/ERS/JRS/ALAT [1]. Indeed, ILD was defined as PPF in the presence of at least two of the following criteria in the preceding year: (a) worsening of respiratory symptoms; (b) HRCT evidence of disease progression; and (c) functional impairment proved by an absolute decline of forced vital capacity (FVC) of ≥5% or diffusing capacity of the lungs for carbon monoxide (DLCO) ≥10%.

### 2.4. Statistical Analysis

Statistical analysis was performed with IBM SPSS Statistics for Windows, Version 30.0 (Armonk, NY, USA: IBM Corp.). Continuous variables were described as mean ± SD and categorical variables as n (%). Between-group comparisons (SjS-ILD vs. ASyS-ILD) used χ^2^ or Fisher’s exact tests for categorical variables and independent-samples *t* tests (or Mann–Whitney U when non-normal on Shapiro–Wilk test) for continuous variables. Longitudinal pulmonary function values (FVC, both mL and percent predicted, and DLCO as a percentage predicted at baseline, 12 and 24 months) were analyzed with linear mixed-effects models including fixed effects for time, group, anti-Ro52kD status, mycophenolate use and their prespecified interactions, obtaining mean estimates, standard error and 95% confidence intervals (95% CI). Survival analysis between study groups was performed using the Kaplan–Meier method with log-rank tests. Time-to-event outcomes (initiation of long-term oxygen therapy, death and progression to PPF) were assessed using Cox proportional-hazards models, estimating size effects as hazard ratios (HRs) with relative 95% CI for anti-Ro52kD within and across subgroups. We prespecified unadjusted analyses as the primary approach, reflecting the study’s descriptive aim and the balance in age and sex between groups (standard mean differences ≤ 50%). To assess robustness, we performed sensitivity analyses adjusted for age and sex. Given the limited number of events and the small sample size, no additional covariates were included to avoid overfitting. Two-sided *p* < 0.05 was considered statistically significant.

## 3. Results

**Figure 1:**
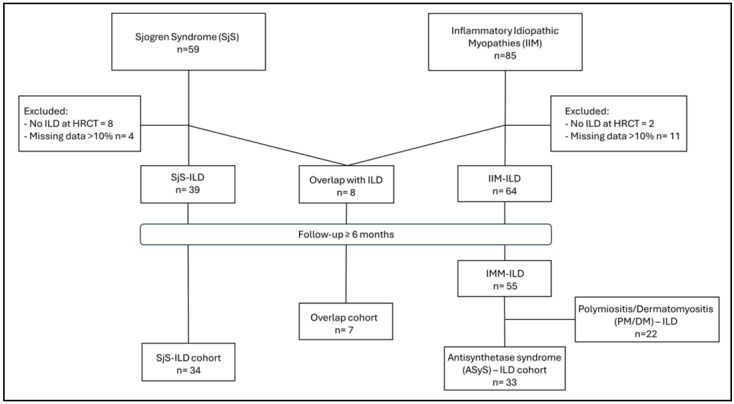
Design of enrollment and selection of study cohorts. Legend: DM: Dermatomyositis; ILD: HRCT: High-Resolution Computed Tomography; interstitial lung disease; PM: Polymyositis.

From the analysis of our clinical electronic database, 67 patients met the inclusion and exclusion criteria proposed, comprising 34 with Sjögren’s Syndrome-associated ILD (SjS-ILD) and 33 with Anti-Synthetase Syndrome-associated ILD (ASyS-ILD); 7 patients classifiable as overlap syndrome were excluded (Figure 1).

Women represented most of the included patients in both cohorts, with no significant difference (76.5% of the SjS-ILD group vs. 69.7% of the ASyS-ILD cohort, *p* = 0.532). Mean age at ILD diagnosis was similar between cohorts, 64.5 ± 1.53 vs. 61.38 ± 1.71 years (*p* = 0.734) in SjS-ILD and ASyS-ILD, respectively. Smoking status distributions showed most SjS-ILD patients had never smoked (70.6%), differing from the ASyS-ILD group where 57.6% had smoking exposure (former plus current smokers). The timing of disease onset significantly differed: ILD as the first manifestation occurred in 55.9% of SjS-ILD vs. 21.2% of ASyS-ILD, whereas concomitant onset was 26.5% vs. 66.7% (global *p* = 0.03). Demographic and clinical characteristics of the study cohorts are displayed in Table 1.

### 3.1. Serological, Clinical and Radiological Characteristics

ANA positivity (≥1:160) was comparable between groups (47.1% vs. 45.4% in SjS-ILD and ASyS-ILD; *p* = 0.895), with a similar prevalence of a speckled pattern (*p* = 0.901). In contrast, the cytoplasmatic pattern was more frequent in ASyS-ILD (24.2%) than in SjS-ILD (2.9%; *p* = 0.005). Anti-Ro52kD positivity was predominant in SjS-ILD, significantly different from the data registered in the ASyS-ILD group (61.8% vs. 27.3%, respectively; *p* = 0.002). Myositis-specific antibodies (MSA) were globally present in the ASyS-ILD cohort with no cases in the SjS-ILD group, with Jo1 as the most frequent single antibody (*n* = 15, 45.45%). In the absence of anti-Jo1 positivity, patients included in the ASyS-ILD cohort were found positive for at least one anti-aminoacyl tRNA-synthetase (ARS) antibody (anti-PL7, anti-PL12, anti-EJ, anti-OJ). Myositis-associated antibodies (MAA), excluding anti-SSA/SSB antibodies, were found in only two cases (5.9%) of SjS-ILD, while in the ASyS-ILD cohort, more than half of patients were positive (54.1%; *p* < 0.001).

As would reasonably be expected, sicca syndrome predominated in SjS-ILD (85.3%) and was uncommon in ASyS-ILD (9.1%; *p* < 0.001), and myositis was more frequent in ASyS-ILD (18.2%) than in SjS-ILD (2.9%; *p* = 0.041). Other clinical manifestations were similar between groups (Table 2), even for conditions potentially specific for ASyS such as arthritis, found in 17.6% vs. 24.2% in SjS-ILD and ASyS-ILD, respectively (*p* = 0.507).

Systemic corticosteroid use was frequent and similar (97.1% vs. 93.9%; *p* = 0.537). Mycophenolate mofetil was prescribed more often in ASyS-ILD (69.7%) than in SjS-ILD (32.3%; *p* = 0.002). Use of other immunosuppressant drugs did not differ materially. In our study cohorts, the number of patients who started antifibrotic therapy with Nintedanib was comparable between groups (8.8% vs. 12.1%; *p* = 0.659).

The overall distribution of HRCT patterns did not show a significant difference between groups (*p* = 0.188). In SjS-ILD, NSIP was most common—38.2% of NSIP and 26.5% of fibrotic NSIP—followed by UIP (8.8%) and probable UIP (11.8%); an NSIP + OP pattern occurred in four cases (11.8%), and LIP was registered only in one patient (2.9%). In ASyS-ILD, fibrotic NSIP (39.4%) and NSIP (18.2%) predominated, with an OP pattern found in four subjects and combined pattern NSIP + OP in five patients (15.2%); UIP and probable UIP covered 6.1% and 9.1% of the cohort, respectively.

Serological, clinical, pharmacological and radiological features of the study groups are summarized in Table 2.

### 3.2. Longitudinal Lung Function Evaluation

**Figure 2:**
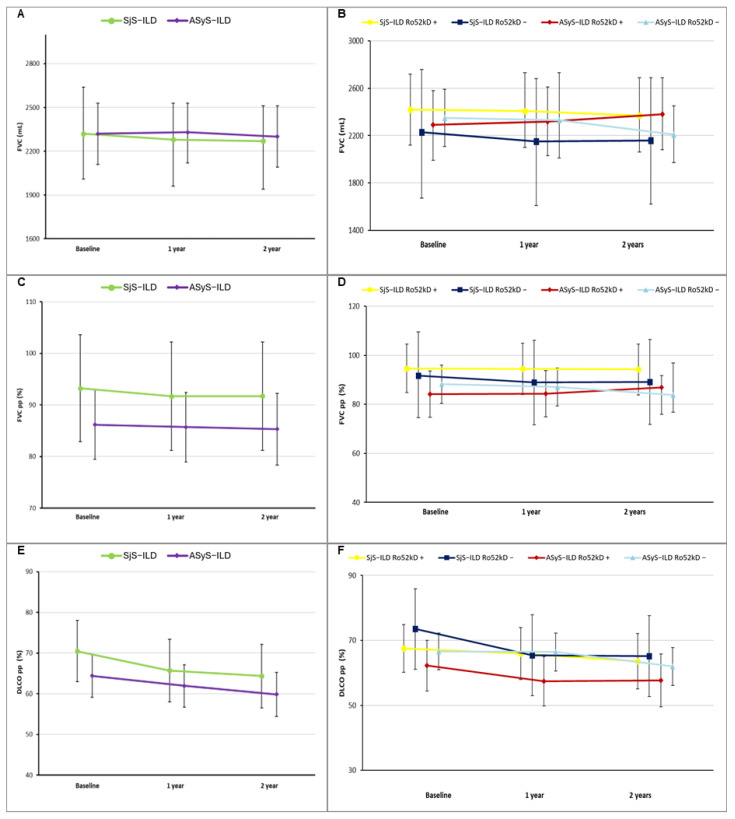
Comparison between longitudinal lung function course adjusted for mycophenolate use in study groups expressed as mean estimates and 95% confidence intervals for (**A**) FVC (mL), (**B**) FVC pp (%) and (**C**) DLCO pp (%) and sub-analysis according to anti-Ro52k status for (**D**) FVC (mL), (**E**) FVC pp (%) and (**F**) DLCO pp (%). *Abbreviations:* SjS-ILD: Sjögren’s Syndrome-associated interstitial lung disease; ASyS-ILD: Anti-Synthetase Syndrome-associated interstitial lung disease; FVC: forced vital capacity; FVC pp: percent-predicted forced vital capacity; DLCO: diffusing capacity of the lungs for carbon monoxide; DLCO pp: percent-predicted diffusing capacity of the lungs for carbon monoxide.

Trajectories of FVC (ml and pp) and DLCO over 24 months are shown in Figure 2. Considering the imbalance in mycophenolate mofetil (MMF) prescription (32.3% in SjS-ILD vs. 69.7% in ASyS-ILD, *p* = 0.002), all mean estimates were adjusted for MMF use, without showing significant differences compared to unadjusted models (mean estimated values are reported in Supplemental Materials, Tables S1 and S2, respectively,). Across the three timepoints (baseline, 1 year, 2 years), mean estimates for FVC and FVC percent predicted (FVCpp) were broadly comparable between SjS-ILD and ASyS-ILD, with no significant differences at any timepoint (Figure 2A,B). Within the SjS-ILD cohort, baseline FVC (2320 mL, 95% CI 2.01–2.64) and FVCpp (93.23%, 95CI% 82.88–103.58) had a mild decline in 2 years, losing around 50 mL and 1.54%, respectively. ASyS-ILD showed a similar trend, with FVC/FVCpp at baseline 2320 mL/86.17% (95% CI2110–2530/79.43–92.91) decreasing up to 2300 mL/85.32 after 2 years. For DLCO, both conditions were comparable at baseline (70.48% vs. 64.40%, respectively, for SjS-ILD and ASyS-ILD), maintaining the same trend across the timeframe considered, showing a similar decline at 2 years (−6.12% in SjS-ILD vs. −4.55% in ASyS-ILD). Model-based mean estimates, standard errors, 95% CIs and *p*-value for comparison between study groups are reported in Supplemental Materials, Tables S1 and S2.

Successively, a sub-analysis was performed on patients clustered according to anti-Ro52kD status, maintaining the adjusted model for mycophenolate use due to differences among subgroups in its utilization (88% in ASyS-ILD anti-Ro52kD+ vs. anti-Ro60kD in ASyS-ILD anti-Ro52kD−%; 40% in Sjögren-ILD anti-Ro52kD+ vs. 27% in Sjögren-ILD anti-Ro52kD−), showing opposite patterns across diseases. In fact, in SjS-ILD, anti-Ro52kD positivity was associated with higher mean values of FVC (both absolute values and pp) than seronegative subjects across the three timepoints considered; however, DLCO was slightly lower at baseline (67.49% vs. 73.48%), but with a decline approximately half that observed in the anti-Ro52kD− subgroup (3.93% vs. 8.31%, respectively).

On the other hand, the ASyS-ILD anti-Ro52kD-positive subgroup showed a non-significant lower estimate for FVC at baseline (2290 mL, 95% CI 1990–2580/84.13%, 95% CI 74.7–93.55 in anti-Ro52kD+ vs. 2350 mL, 95% CI 2110–2590/88.21%, 95CI% 80.47–95.95 in anti-Ro52kD−, *p* = 0.74/0.51), but FVC in the anti-Ro52kD+ subgroup improved over time, with higher values at 2 years compared with the anti-Ro52− subgroup (2380 mL/86.82% vs. 2210 mL/83.82%, *p* = 0.49/*p* = 0.64).

Nevertheless, DLCO was lower in ASyS-ILD patients positive for anti-Ro52kD at all timepoints, showing a similar rate of decline after 2 years (4.5% in anti-Ro52kD+ vs. 4.63% in anti-Ro52kD−). Interestingly, by comparing subgroups of anti-Ro52kD-positive patients in both conditions, no significant difference was found for FVC and DLCO at any timepoint. Finally, anti-Ro52kD− patients had comparable lung function decline over time regardless of the underlying disease, whereas SjS-ILD showed greater stability across 2 years for FVC (loss of 70 mL/2.69% compared to 140 mL/4.39% in ASyS-ILD); however, DLCO declined 8.31% in the same timeframe, more than double the decrease in ASyS-ILD (4.63%). All mean estimates, standard errors, 95% CIs and *p*-values comparing subgroups within underlying conditions or anti-Ro52kD status are fully described in Supplementary Materials, Tables S3–S6.

### 3.3. Mortality and Time-to-Event Risk Estimates

**Figure 3:**
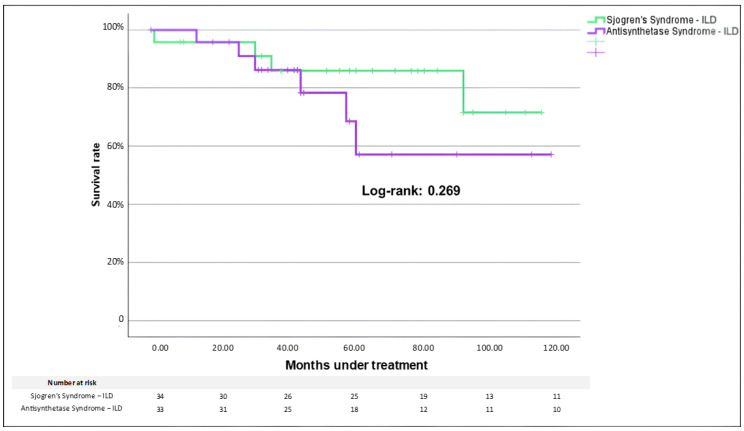
Comparison of Kaplan–Meier survival curve between Sjögren Syndrome-ILD and Anti-Synthetase Syndrome-ILD groups. Legend: ILD: interstitial lung disease.

During follow-up, a need for supplemental oxygen therapy occurred in 38.2% of SjS-ILD and 39.4% of ASyS-ILD (*p* = 0.92). Fibrotic progression according to PPF proposed criteria was similarly found in study cohorts (23.5% vs. 32.3%, SjS-ILD and ASyS-ILD, respectively; *p* = 0.412). During the observation period, comparable mortality was registered as slightly higher but not significantly different in ASyS-ILD (18.2%) than in SjS-ILD (11.8%, *p* = 0.349). Kaplan–Meier curves did not show a difference between cohorts (Log-rank *p*-value: 0.269, Figure 3). In both cohorts, 50% (*n* = 2 in SjS-ILD vs. *n* = 3 in ASyS-ILD) of registered deaths were accounted for by the worsening of the respiratory condition.

**Figure 4:**
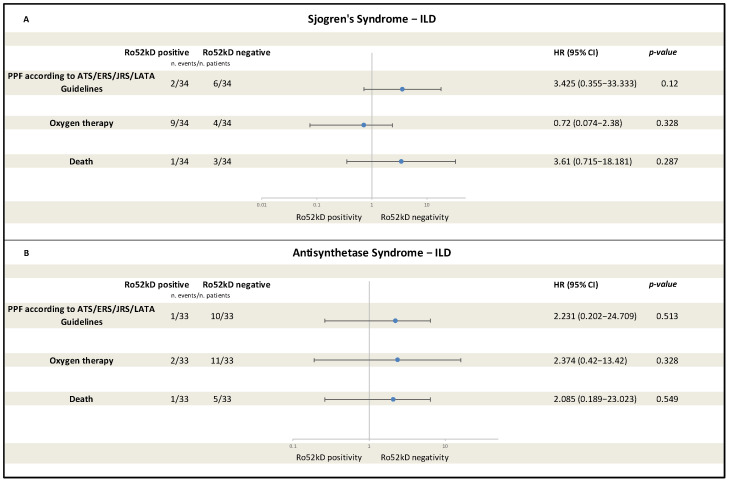
Forest plot of unadjusted hazard ratios (HRs) comparing anti-Ro52kD+ versus anti-Ro52kD− patients for three time-to-event outcomes in (**A**) Sjogren’s Syndrome-ILD and (**B**) Anti-Synthetase Syndrome-ILD: progression to PPF (defined according to ATS/ERS/JRS/ALAT criteria), initiation of oxygen therapy, and all-cause death. Event counts (events/total) for anti-Ro52kD+ and anti-Ro52kD− groups are shown beside each outcome. *Abbreviations:* ILD: interstitial lung disease; HR: hazard ratio; CI: confidence interval; PPF: progressive pulmonary fibrosis; ATS: American Thoracic Society; ERS: European Respiratory Society; JRS: Japanese Respiratory Society; ALAT: Asociación Latinoamericana de Tórax.

Cox models estimated unadjusted HRs for anti-Ro52kD status in both cohorts. In both cohorts, anti-Ro52kD positivity was not significantly associated with occurrence of any of the three time-to-event outcomes selected for the analysis (meeting PPF criteria, initiation of oxygen therapy, death). Forest plots including HRs for selected outcomes are displayed in Figure 4. In SjS-ILD, progression to PPF and death were both favored by anti-Ro52kD negativity (HR = 3.43, 95% CI 0.36–33.33, *p* = 0.12 and HR= 3.61, 95% CI 0.72–18.18, *p* = 0.29, respectively), but no significant difference was demonstrated. In the same way, oxygen therapy was not related to anti-Ro52kD status, although it showed a weak association with antibody positivity (HR = 0.72, 95% CI 0.07–2.38; *p* = 0.33) (Figure 4A). In ASyS-ILD, the corresponding HRs for anti-Ro52kD negativity were 2.23 for evolution to PPF (95% CI 0.20–24.71; *p* = 0.51), 2.37 for need of oxygen therapy (95% CI 0.42–13.42; *p* = 0.33) and 2.09 for death (95% CI 0.19–23.02; *p* = 0.55) (Figure 4B). Despite all the outcomes showing a tendency related to a seronegative profile, all confidence intervals were wide and crossed unity, indicating lack of statistical significance, probably related to the small sample size and the fact that few events were included. 

In age- and sex-adjusted sensitivity analyses, estimates were attenuated but directionally consistent (Supplemental Materials, Figure S1), although confidence intervals widened, reflecting limited power. In the SjS-ILD cohort, adjustment yielded smaller HRs with persistent wide CIs (PPF: HR 1.30, 95% CI 0.26–6.41; need of oxygen therapy: HR 1.75, 95% CI 0.19–16.17; death: HR 2.53, 95% CI 0.23–28.41). In ASyS-ILD, point estimates were attenuated for PPF (HR 1.24, 95% CI 0.22–6.99) and oxygen (HR 0.42, 95% CI 0.08–2.15), while the HR for death increased but remained very imprecise (HR 6.19, 95% CI 0.62–62.17).

## 4. Discussion

Similarities between SjS and ASyS were present since patient enrolment. Approximately 10% of patients were excluded because they met classification criteria for both conditions. SjS overlapped with other systemic autoimmune conditions in up to 60% of cases, and myositis is reported in about 15% of pSS patients [28]. However, a specific association between SjS and ASyS is reported only in few cases. Other retrospective cohorts reported that roughly one third of SjS patients with myositis showed histological findings for inclusion body myositis whereas other patients satisfied Bohan & Peter criteria for Polymyositis and Dermatomyositis [28,29].

Smoking is a well-recognized risk factor for development of ILD, and the proportion of smokers is greater in ASyS. This may reflect the role of smoking in triggering lung immunity, well-established in the citrullination of proteins in RA but also reported for anti-Jo1, the most common ASA, and conversely not proven for anti-SSA that target ubiquitous antigens [30].

ILD was the first manifestation of SjS in most patients, showing a significant difference compared with ASyS. By contrast, the development of ILD after a specific diagnosis of either condition is reported in about 20% of cases. As reported in other studies [9,11], this is well-documented, but the magnitude of this proportion may be influenced by selection bias, considering that we enrolled patients in a respiratory unit. In any case, diagnosis can be difficult in patients without other suggestive signs.

The differential diagnosis may also not be straightforward: from a clinical perspective, although sicca syndrome and myositis were mainly reported in SjS and ASyS, respectively, it is noteworthy that other clinical features deemed useful for diagnosing ASyS, such as RP, unexplained fever, or inflammatory arthritis—part of the classic ASyS triad [8]—did not differ between the two groups. In the same way, radiological patterns of ILD were largely similar: only one patient showed a radiological pattern of LIP, uncommon but highly specific for SjS; other radiological patterns were substantially comparable across groups.

The most important distinction lies in serology. Except for one ASyS patient, all were seropositive for ASA, which were not detected in SjS patients, whereas about 70% of SjS patients were positive for anti-SSA, mostly anti-Ro52kD (61.8%). This antibody, classified as a MAA, was also present in a non-negligible proportion of ASyS patients (27.3%), as expected by previous studies [15]. The lower proportion in our cohort should be interpreted considering the exclusion of seven patients classified as overlap condition SjS-ASyS due to the presence of sicca syndrome proved by Schirmer Test, ILD, ASA and anti-Ro52kD. Specific seropositivity can also explain the difference in the ANA pattern, with a higher frequency of a cytoplasmic pattern in ASyS, consistent with ASA positivity [31].

The conditions were also similar regarding disease progression. Trajectories of FVC and proportions of respiratory failure and death were comparable between the two study cohorts. This finding might be the expression of an early use of immunosuppressive drugs: in our cohort, a greater proportion of ASyS patients were treated with mycophenolate mofetil, which is the first-line treatment for CTD-ILD but also useful for the management of active myositis [32,33]. The use of MMF in our cohort was not associated with different clinical or functional parameters; however, some ASyS patients may have received the drug also due to their muscle involvement, given that myositis, as expected, was mainly reported in the ASyS cohort.

In our study, positivity for anti-Ro52kD is associated with lower baseline values of FVC and DLCO in the ASyS cohort, but these differences diminished with treatment, resulting in similar functional parameters at the end of follow-up regardless of anti-Ro52kD status. Comparing anti-Ro52kD+ patients across SjS and ASyS, no differences were noted in terms of mortality, disease progression or FVC. However, DLCO remained lower through the whole follow-up in ASyS patients. A possible explanation could be concomitant myositis—typical of ASyS, at least sub-clinically—which is able to reduce DLCO without indicating greater lung damage [34], as well as similar causes such as vascular involvement or hypoventilation [35]. Although anti-Ro52kD is generally associated with the presence of ILD and potential fibrotic and/or rapid progression [36,37], our findings showed similar rates of progression and prognosis in patients with and without anti-ro52kD positivity in both SjS and ASyS cohorts. This might reflect the limited sample size, even though it should be considered that the absence of anti-SSA is described as an independent factor of worse outcomes in ILD-SjS [38]. Moreover, in our ASyS cohort, a significant proportion of patients negative for anti-Ro52kD showed positivity for non-anti-Jo1 ASA, which are also associated with a poorer ILD prognosis [39].

This study has some limitations. First, the retrospective design with data obtained from routine clinical records, rather than collected under a prospective research protocol, was missing some clinical information (e.g., occurrence of adverse events related to pharmacological treatment). Second, the small sample size limits its statistical power, resulting in wide confidence intervals and a higher risk of type II error; furthermore, residual confounding cannot be excluded. Moreover, the enrollment of ASyS patients was conducted according to the Connors Criteria, which are not validated [20]. Connors criteria are currently widely used for ASyS classification in clinical research, but in any case, our ASyS will also be enrolled as ASyS in line with the upcoming validated criteria [8]. Seropositivity for ASA was evaluated with immunoblotting, without confirmation through immunoprecipitation. Despite autoimmunity being confirmed on annual testing, the rate of false positivity with immunoblotting should be taken into account. To improve specificity of our testing, we considered true positive autoantibodies with at least a medium titer and/or the presence of appropriate pattern in ANA positivity (for example, cytoplasmic in ASA positivity) [40,41,42]. HRCT were evaluated by a single expert radiologist; therefore, the inter-observer agreement was not assessed. Finally, the greater proportion of patients treated with MMF in the ASyS cohort could have mitigated the progression of lung damage in these patients, considering that MMF is a first-line treatment for CTD-ILD. These factors, together with the monocentric setting, may restrict the generalizability of our findings. Accordingly, the results should be interpreted as exploration and warrant confirmation in prospective and multicenter studies with standardized data collection.

## 5. Conclusions

In conclusion, patients with ILD associated with ASyS and SjS share several features. Despite some differences in the prevalence of myositis and sicca syndrome, other accompanying manifestations such as inflammatory arthritis, RP and fever were comparable. Similarities were also present in the prevalence of different radiological patterns of ILD, except for LIP pattern, which is rare but highly specific for SjS. The main difference was serological, with an obvious greater prevalence of ASA in ASyS, but with a notable proportion of ASyS patients reporting positivity for anti-Ro52kD, the prevalent autoantibody detected in our ILD-SjS cohort. Finally, even disease progression and prognosis were similar across study cohorts. These observations raise the possibility that ILD-SjS cohorts may include some patients with unrecognized or undetectable ASAs. In this context, further prospective studies altogether with the upcoming ASyS classification criteria may be useful to identifying these patients and prompt deeper serological assessment to advance our knowledge of ASAs.

## Figures and Tables

**Table 1:** Demographic characteristics of study cohorts.

	Sjogren’s Syndrome-ILD	Anti-Synthetase Syndrome-ILD	*p*-Value
	(*n* = 34)	(*n* = 33)	
Sex (female)	26 (76.5%)	23 (69.7%)	0.532
Age at diagnosis of ILD (years)	64.5 ± 1.53	61.38 ± 1.71	0.734
**Smoking history**			0.053
Never	24 (70.6%)	14 (42.4%)	
Former	8 (23.5%)	17 (51.5%)	
Current	2 (5.9%)	2 (6.1%)	
Smoking exposure (pack/years)	15.33 ± 2.76	21.10 ± 2.88	0.289
Age at smoking initiation	17.41 ± 3.22	18.71 ± 4.63	0.772
BMI (kg/m^2^)	27.45 ± 0.65	28.75 ± 2.60	0.49
**Disease onset**			0.03
ILD as first manifestation	19 (55.9%)	7 (21.2%)	
SjS/ASyS first, ILD then	6 (17.6%)	4 (12.1%)	
Concomitant onset	9 (26.5%)	22 (66.7%)	
Supplemental oxygen therapy	13 (38.2%)	13 (39.4%)	0.92
Evolution to PPF	8 (23.5%)	11 (32.3%)	0.412
Death during follow-up	4 (11.8%)	6 (18.2%)	0.349

*Abbreviations*: ILD: ASyS: Anti-Synthetase syndrome BMI: body mass index; interstitial lung disease; PPF: progressive pulmonary fibrosis. SjS: Sjogren’s Syndrome.

**Table 2:** Serological, clinical and radiological features in study groups.

	SjS-ILD	ASyS-ILD	*p*-Value
	(*n* = 34)	(*n* = 33)	
**Antibody positivity**			
ANA ≥ 1:160	16 (47.1%)	15 (45.4%)	0.895
ANA—Speckled pattern	8 (23.5%)	7 (21.2%)	0.901
ANA—Cytoplasmatic pattern	1 (2.9%)	8 (24.2%)	0.005
Anti-SSA/Ro52kD	21 (61.8%)	9 (27.3%)	0.002
Anti-SSA/Ro60kD	2 (5.9%)	/	0.151
Anti-SSB/La	2 (5.9%)	/	0.157
Myositis-specific antibodies	/	32 (96.7%)	<0.001
Myositis-associated antibodies *	2 (5.9%)	18 (54.5%)	<0.001
**Clinical manifestations**			
Sicca syndrome	29 (85.3%)	3 (9.1%)	<0.001
Myositis	1 (2.3%)	6 (18.2%)	0.041
Arthritis	6 (17.6%)	8 (24.2%)	0.507
Myalgia	2 (5.9%)	6 (18.2%)	0.121
Fever of unknown origin	4 (11.8%)	8 (24.2%)	0.183
Raynaud’s Phenomenon	7 (20.6%)	8 (24.2%)	0.521
			
**Pharmacological treatment**			
Corticosteroids	33 (97.1%)	31 (93.9%)	0.537
Mycophenolate mofetil	11 (32.3%)	23 (69.7%)	0.002
Azathioprine	9 (26.5%)	10 (30.3%)	0.728
Rituximab	1 (2.9%)	1 (3.03%)	0.983
Cyclophosphamide	/	2 (6.1%)	0.145
Methotrexate	5 (14.7%)	3 (9.1%)	0.479
Hydroxychloroquine	9 (26.5%)	5 (15.2)	0.255
Nintedanib	3 (8.8%)	4 (12.1%)	0.659
			
**HRCT pattern**			0.188
UIP	3 (8.8%)	2 (6.1%)	
UIPp	4 (11.8%)	3 (9.1%)	
Fibrotic NSIP	9 (26.5%)	13 (39.4%)	
NSIP	13 (38.2%)	6 (18.2%)	
NSIP + OP	4 (11.8%)	5 (15.2%)	
OP	/	4 (12.1%)	
LIP	1 (2.9%)	/	
			

* Anti-SSA/SSB excluded. *Abbreviations:* ANA: antinuclear antibody; ASyS-ILD: Anti-Synthetase syndrome-associated interstitial lung diseases; HRCT: High-Resolution Computed Tomography; LIP: Lymphocytic Interstitial Pneumonia; NSIP: Nonspecific Interstitial Pneumonia; OP: Organizing Pneumonia; SjS-ILD: Sjogren’s Syndrome-associated interstitial lung disease; UIP: usual interstitial Pneumonia; UIPp: usual interstitial pneumonia probable.

## Data Availability

The data presented in this study are available on request from the corresponding author due to legal and ethical reasons.

## Supplementary Materials

The following supporting information can be downloaded at: https://www.mdpi.com/article/10.3390/medicina61112044/s1, Table S1. Comparison of lung function at different timepoints between Sjogren’s Syndrome and Anti-Synthetase Syndrome. Table S2. Comparison of lung function adjusted for mycophenolate mofetile use at different timepoints between Sjogren’s Syndrome and Anti-Synthetase Syndrome. Table S3. Comparison of lung function adjusted for mycophenolate mofetile use at different timepoints between each condition according to Ro52k status. Table S4. Comparison of unadjusted lung function at different timepoints between each condition according to Ro52k status. Table S5. Comparison of lung function adjusted for mycophenolate mofetile use at different timepoints within each condition according to Ro52k status. Table S6. Comparison of unadjusted lung function at different timepoints within each condition according to Ro52k status. Table S7. Comparison of lung function in Anti-synthetase-ILD positive/negative for Ro52kD according to anti-Jo1 or other RSA at different timepoints. Figure S1. Forest plot of hazard ratios (HRs) adjusted for age and sex comparing Ro52kD-positive versus Ro52kD-negative patients for three time-to-event outcomes in (A) Sjogren’s Syndrome-ILD and (B) Antisynthetase Syndrome-ILD: progression to PPF (defined according to ATS/ERS/JRS/ALAT criteria), initiation of oxygen therapy, and all-cause death.

## Abbreviations

ANA: Antinuclear Antibodies, ASA: Anti-Synthetase Antibody; ASyS: Anti-Synthetase Syndrome; CTD: Connective Tissue Disease; DLCO: Diffusion Lung Capacity for Carbon Monoxide (DLCO); ENA: Extractable Nuclear Antibodies; FVC: Forced Vital Capacity IIM: Idiopathic Inflammatory Myopathy; ILD: Interstitial Lung Disease; IPAF: Interstitial Pneumonia with Autoimmune Features; HRCT: High-Resolution Computed Tomography; IPF: Idiopathic Pulmonary Fibrosis; LIP: Lymphocytic Interstitial Pneumonia; MAA: Myositis-Associated Antibodies; MSA: Myositis-Specific Antibodies; NSIP: Nonspecific Interstitial Pneumonia; NVC: Nailfold Videocapillaroscopy; OP: Organizing Pneumonia; PFP: Progressive-Fibrosing Phenotype; SjS: Sjögren’s Syndrome; RA: Rheumatoid Arthritis; RP: Raynaud’s Phenomenon; SSc: Systemic Sclerosis; UIP: Usual Interstitial Pneumonia
