# Supplementary material for: Interstitial Lung Disease Secondary to Sjogren’s Syndrome and Antisynthetase Syndrome: Converging Disease Trajectories
Source: Medicina (Kaunas). 2025 Nov 15;61(11):2044. doi: 10.3390/medicina61112044 (PMC12654823; doi:10.3390/medicina61112044)
Supplement: Supplementary file 1 [file medicina-61-02044-s001.zip › medicina-3949380-supplementary.pdf]

Supplemental Materials

Table S1. Comparison of lung function at different timepoints between Sjogren's Syndrome and Anti-Synthetase Syndrome. .

	Baseline			1 year follow-up			2 years follow-up		
	**SjS-ILD (n = 34)**	**ASyS-ILD (n = 33)**	***p*-value**	**SjS-ILD (n = 34)**	**ASyS-ILD (n = 33)**	***p*-value**	**SjS-ILD (n = 34)**	**ASyS-ILD (n = 33)**	***p*-value**
**FVC (L)**	2.32±0.18 (95%CI 2.08-2.56)	2.44±0.13 (95%CI 2.17-2.71)	0.494	2.24±0.18 (95%CI 2.00-2.48)	2.42±0.13 (95%CI 2.14-2.69)	0.322	2.22±0.18 (95%CI 1.98-2.46)	2.32±0.14 (95%CI 2.17-2.71)	0.598
**FVC pp (%)**	93.25±3.80 (95%CI 85.54-100.95)	87.34±4.71 (95%CI 77.74-96.93)	0.333	90.54±3.9 (95%CI 82.66-98.42)	86.32±4.78 (95%CI 76.61-96.04)	0.496	90.46±3.87 (95CI% 82.63-98.29)	84.04±4.85 (95%CI 74.19-93.88)	0.304
**DLCO pp (%)**	68.94±3.54 (95%CI 61.78.-76.09)	63.59±3.33 (95%CI 56.83-70.34)	0.275	68.47±3.59 (95%CI 61.23-75.71)	62.09±3.45 (95%CI 55.11-69.07)	0.204	67.48±3.58 (95%CI 60.25-74.73)	55.96±3.52 (95%CI 48.86-63.06)	0.024

Abbreviations: SjS-ILD: Sjögren's syndrome associated interstitial lung disease; ASyS-ILD: anti-synthetase syndrome–associated interstitial lung disease; FVC: forced vital capacity; FVC pp: percent-predicted forced vital capacity; DLCO: diffusing capacity of the lung for carbon monoxide; ; DLCO pp: percent predicted diffusing lung capacity for carbon monoxide; CI = confidence interval.

Table S2. Comparison of lung function adjusted for mycophenolate mofetil use at different timepoints between Sjogren's Syndrome and Anti-Synthetase Syndrome. .

	Baseline			1 year follow-up			2 years follow-up		
	**SjS-ILD (n = 34)**	**ASyS-ILD (n = 33)**	***p*-value**	**SjS-ILD (n = 34)**	**ASyS-ILD (n = 33)**	***p*-value**	**SjS-ILD (n = 34)**	**ASyS-ILD (n = 33)**	***p*-value**
**FVC (L)**	2.32±0.16 (95%CI 2.01-2.64)	2.32±0.10 (95%CI 2.11-2.53)	0.98	2.28±0.16 (95%CI 1.96-2.60)	2.33±0.10 (95%CI 2.12-2.53)	0.56	2.27±0.16 (95%CI 1.94-2.59)	2.30±0.10 (95%CI 2.09-2.51)	0.86
**FVC pp (%)**	93.23±5.17 (95%CI 82.88-103.58)	86.17±3.37 (95%CI 79.43-92.91)	0.26	91.69±5.25 (95%CI 81.19-102.18)	85.71±3.38 (95%CI 78.94-92.47)	0.34	91.72±3.49 (95CI% 81.22-102.21)	85.32±3.49 (95CI% 78.35-92.23)	0.31
**DLCO pp (%)**	70.48±3.76 (95%CI 62.96.-78.01)	64.40±2.64 (95%CI 59.12-69.68)	0.19	65.70±3.85 (95%CI 58.01-73.39)	61.93±2.62 (95%CI 56.68-67.17)	0.42	64.36±3.92 (95%CI 56.54-72.19)	59.85±2.8 (95%CI 54.40-65.29)	0.35

Abbreviations: SjS-ILD: Sjögren's syndrome associated interstitial lung disease; ASyS-ILD: anti-synthetase syndrome–associated interstitial lung disease; FVC: forced vital capacity; ppFVC: percent-predicted forced vital capacity; DLCO: diffusing capacity of the lung for carbon monoxide; ; DLCO pp: percent predicted diffusing lung capacity for carbon monoxide; CI = confidence interval.

Table S3. Comparison of lung function adjusted for mycophenolate mofetil use at different timepoints between each condition according to anti-Ro52k status. .

	Anti-Ro52kD positive

	Baseline			1 year follow-up			2 years follow-up		
	**SjS-ILD (n = 21)**	**ASyS-ILD (n = 9)**	***p*-value**	**SjS-ILD (n = 21)**	**ASyS-ILD (n = 9)**	***p*-value**	**SjS-ILD (n = 21)**	**ASyS-ILD (n = 9)**	***p*-value**
**FVC (L)**	2.42±0.15 (95%CI 2.12-2.72)	2.29±0.15 (95%CI 1.99-2.58)	0.54	2.41±0.16 (95%CI 2.10-2.73)	2.32±0.14 (95%CI 2.03-2.61)	0.67	2.37±0.16 (95%CI 2.06-2.69)	2.38±0.15 (95%CI 2.08-2.69)	0.96
**FVC pp (%)**	94.69±4.92 (95%CI 84.83-104.54)	84.13±4.71 (95%CI 74.70-93.55)	0.32	94.49±5.24 (95%CI 84.00-104.89)	84.34±4.73 (95%CI 74.89-93.78)	0.14	94.25±5.24 (95%CI 83.81-104.67)	86.82±5.02 (95%CI 75.98-91.65)	0.31
**DLCO pp (%)**	67.49±3.67 (95%CI 60.15-74.83)	62.21±3.93 (95%CI 54.40-70.02)	0.33	65.98±4.02 (95%CI 57.99-73.98)	57.44±3.79 (95%CI 49.85-65.023)	0.12	63.56±4.29 (95%CI 55.06-72.06)	57.71±3.79 (95%CI 49.57-65.84)	0.32

	Anti-Ro52kD negative								
	**Baseline**			**1 year follow-up**			**2 years follow-up**		
	**SjS-ILD (n = 13)**	**ASyS-ILD (n = 24)**	***p*-value**	**SjS-ILD (n = 13)**	**ASyS-ILD (n = 24)**	***p*-value**	**SjS-ILD (n = 13)**	**ASyS-ILD (n = 24)**	***p*-value**
**FVC (L)**	2.23±0.26 (95%CI 1.67-2.76)	2.35±0.12 (95%CI 2.11-2.59)	0.67	2.15±0.27 (95%CI 1.61-2.68)	2.33±0.12 (95%CI 2.01-2.73)	0.37	2.16±0.27 (95%CI 1.62-2.69)	2.21±0.12 (95%CI 1.97-2.45)	0.86
**FVC pp (%)**	91.77±8.64 (95%CI 74.49-109.54)	88.21±3.87 (95%CI 80.47-95.95)	0.71	88.93±8.64 (95%CI 71.64-106.21)	87.08±3.89 (95%CI 79.29-94.86)	0.81	89.18±8.64 (95%CI 71.90-106.47)	83.82±3.92 (95%CI 76.81-96.82)	0.57
**DLCO pp (%)**	73.48±6.22 (95%CI 61.05-85.91)	66.59±2.83 (95%CI 60.92-72.26)	0.32	65.42±6.22 (95%CI 52.99-77.85)	66.42±2.93 (95%CI 60.57-72.26)	0.88	65.17±6.22 (95%CI 52.74-77.59)	61.97±2.93 (95%CI 56.14-67.83)	0.64

Abbreviations: SjS-ILD: Sjögren's syndrome associated interstitial lung disease; ASyS-ILD: anti-synthetase syndrome–associated interstitial lung disease; FVC: forced vital capacity; FVC pp: percent-predicted forced vital capacity; DLCO: diffusing capacity of the lung for carbon monoxide; ; DLCO pp: percent predicted diffusing lung capacity for carbon monoxide; CI = confidence interval.

Table S4. Comparison of unadjusted lung function at different timepoints between each condition according to anti-Ro52k status. .

	Anti-Ro52kD positive								
	**Baseline**			**1 year follow-up**			**2 years follow-up**		
	**SjS-ILD (n = 21)**	**ASyS-ILD (n = 9)**	***p*-value**	**SjS-ILD (n = 21)**	**ASyS-ILD (n = 9)**	***p*-value**	**SjS-ILD (n = 21)**	**ASyS-ILD (n = 9)**	***p*-value**
**FVC (L)**	2.36±0.16 (95%CI 2.02-2.69)	2.21±0.19 (95%CI 1.79-2.64)	0.562	2.39±0.16 (95%CI 2.05-2.74)	2.27.18±8.44 (95%CI 1.85-2.69)	0.623	2.28±0.16 (95%CI 1.94-2.62)	2.36±0.19 (95%CI 1.93-2.78)	0.757
**FVC pp (%)**	94.11±4.76 (95%CI 84.23-103.99)	75.37±9.29 (95%CI 2.45-2.82)	0.095	95.81±4.83 (95%CI 85.81-105.82)	77.57±9.30 (95%CI 56.38-98.76)	0.105	91.87±4.83 (95%CI 81.87-101.87)	82.12±9.54 (95%CI 60.70-103.54)	0.376
	69.46±4.67	53.17±3.90	0.011	71.81±4.68	45.86±3.66	<0.001	70.82±4.69	47.20±4.22	0.001

DLCO pp (%)	(95%CI 59.87-79.05)	(95%CI 44.78-61.55)		(95%CI 62.33-81.28)	(95%CI 37.95-53.76)		(95%CI 61.22-80.42)	(95%CI 38.16-56.23)	

	Anti-Ro52kD negative								
	Baseline			1 year follow-up			2 years follow-up		
	SjS-ILD (n = 13)	ASyS-ILD (n = 24)	*p*-value	SjS-ILD (n = 13)	ASyS-ILD (n = 24)	*p*-value	SjS-ILD (n = 13)	ASyS-ILD (n = 24)	*p*-value
**FVC (L)**	2.16±0.17 (95%CI 1.77-2.55)	2.52±0.17 (95%CI 2.16-2.88)	0.151	1.88±0.18 (95%CI 1.49-2.28)	2.48±0.18 (95%CI 2.12-2.85)	0.024	2.07±0.17 (95%CI 1.68-2.46)	2.32±0.18 (95%CI 1.95-2.69)	0.325
**FVC pp (%)**	86.15±6.39 (95%CI 72.22-100.09)	92.14±5.34 (95%CI 81.01-103.21)	0.478	75.82±6.58 (95%CI 61.61-90.03)	90.07±5.45 (95%CI 78.81-101.33)	0.106	83.99±6.39 (95%CI 70.11-97.87)	85.83±5.47 (95%CI 74.52-97.14)	0.829
**DLCO pp (%)**	65.13±5.73 (95%CI 52.42-77.85.)	67.08±3.84 (95%CI 59.17-74.99)	0.781	58.33±5.96 (95%CI 45.27-71.39)	68.57±4.08 (95%CI 60.24-76.88)	0,170	57.58±5.73 (95%CI 44.86-70.29)	59.24±4.08 (95%CI 50.92-67.57)	0.815

Abbreviations: SjS-ILD: Sjögren's syndrome associated interstitial lung disease; ASyS-ILD: anti-synthetase syndrome–associated interstitial lung disease; FVC: forced vital capacity; FVC pp: percent-predicted forced vital capacity; DLCO: diffusing capacity of the lung for carbon monoxide; ; DLCO pp: percent predicted diffusing lung capacity for carbon monoxide; CI = confidence interval.

Table S5. Comparison of lung function adjusted for mycophenolate mofetile use at different timepoints within each condition according to anti-Ro52k status.

	Anti-synthetase syndrome - ILD								
	Baseline			1 year follow-up			2 years follow-up		
	Anti-Ro52kD+ (n = 9)	Anti-Ro52kD- (n = 24)	*p*-value	Anti-Ro52kD+ (n = 9)	Ant-Ro52kD- (n = 24)	*p*-value	Anti-Ro52kD + (n = 9)	Anti-Ro52kD- (n = 24)	*p*-value
**FVC (L)**	2.29±0.15 (95%CI 1.99-2.58)	2.35±0.12 (95%CI 2.11-2.59)	0.74	2.32±0.14 (95%CI 2.03-2.61)	2.33±0.12 (95%CI 2.01-2.73)	0.98	2.38±0.15 (95%CI 2.08-2.69)	2.21±0.12 (95%CI 1.97-2.45)	0.49
**FVC pp (%)**	84.13±4.71 (95%CI 74.70-93.55)	88.21±3.87 (95%CI 80.47-95.95)	0.51	84.34±4.73 (95%CI 74.89-93.78)	87.08±3.89 (95%CI 79.29-94.86)	0.64	86.82±5.02 (95%CI 75.98-91.65)	83.82±3.92 (95%CI 76.81-96.82)	0.64
**DLCO pp (%)**	62.21±3.93 (95%CI 54.40-70.02)	66.59±2.83 (95%CI 60.92-72.26)	0.37	57.44±3.79 (95%CI 49.85-65.023)	66.42±2.93 (95%CI 60.57-72.26)	0.06	57.71±3.79 (95%CI 49.57-65.84)	61.97±2.93 (95%CI 56.14-67.83)	0.40

	Sjogren's Syndrome - ILD								
	Baseline			1 year follow-up			2 years follow-up		
	Anti-Ro52kD+ (n = 21)	Anti-Ro52kD- (n = 13)	*p*-value	Anti-Ro52kD+ (n = 21)	Anti-Ro52kD- (n = 13)	*p*-value	Anti-Ro52kD+ (n = 21)	Anti-Ro52kD- (n = 13)	*p*-value
**FVC (L)**	2.42±0.15 (95%CI 2.12-2.72)	2.23±0.26 (95%CI 1.67-2.76)	0.53	2.41±0.16 (95%CI 2.10-2.73)	2.15±0.27 (95%CI 1.61-2.68)	0.26	2.37±0.16 (95%CI 2.06-2.69)	2.16±0.27 (95%CI 1.62-2.69)	0.48

**FVC pp (%)**	94.69±4.92 (95%CI 84.83-104.54)	91.77±8.64 (95%CI 74.49-109.54)	0.82	94.49±5.24 (95%CI 84.00-104.89)	88.93±8.64 (95%CI 71.64-106.21)	0.52	94.25±5.24 (95%CI 83.81-104.67)	89.18±8.64 (95%CI 71.90-106.47)	0.62
**DLCO pp (%)**	67.49±3.67 (95%CI 60.15-74.83)	73.48±6.22 (95%CI 61.05-85.91)	0.41	65.98±4.02 (95%CI 57.99-73.98)	65.42±6.22 (95%CI 52.99-77.85)	0.94	63.56±4.29 (95%CI 55.06-72.06)	65.17±6.22 (95%CI 52.74-77.59)	0.83

Abbreviations: SjS-ILD: Sjögren's syndrome associated interstitial lung disease; ASyS-ILD: anti-synthetase syndrome–associated interstitial lung disease; FVC: forced vital capacity; FVC pp: percent-predicted forced vital capacity; DLCO: diffusing capacity of the lung for carbon monoxide; ; DLCO pp: percent predicted diffusing lung capacity for carbon monoxide; CI = confidence interval.

Table S6. Comparison of unadjusted lung function at different timepoints within each condition according to Ro52k status.

	**Anti-synthetase syndrome - ILD**								
	**Baseline**			**1 year follow-up**			**2 years follow-up**		
	**Anti-Ro52kD+ (n = 9)**	**Anti-Ro52kD- (n = 24)**	***p*-value**	**Anti-Ro52kD+ (n = 9)**	**Anti-Ro52kD- (n = 24)**	***p*-value**	**Anti-Ro52kD+ (n = 9)**	**Anti-Ro52kD- (n = 24)**	***p*-value**
**FVC (L)**	2.21±0.19 (95%CI 1.79-2.64)	2.52±0.17 (95%CI 2.16-2.88)	0.238	2.27.18±8.44 (95%CI 1.85-2.69)	2.48±0.18 (95%CI 2.12-2.85)	0.422	2.36±0.19 (95%CI 1.93-2.78)	2.32±0.18 (95%CI 1.95-2.69)	0.879
**FVC pp (%)**	75.37±9.29 (95%CI 2.45-2.82)	92.14±5.34 (95%CI 81.01-103.21)	0.139	77.57±9.30 (95%CI 56.38-98.76)	90.07±5.45 (95%CI 78.81-101.33)	0.265	82.12±9.54 (95%CI 60.70-103.54)	85.83±5.47 (95%CI 74.52-97.14)	0.74
**DLCO pp (%)**	53.17±3.90 (95%CI 44.78-61.55)	67.08±3.84 (95%CI 59.17-74.99)	0.016	45.86±3.66 (95%CI 37.95-53.76)	68.57±4.08 (95%CI 60.24-76.88)	<0.001	47.20±4.22 (95%CI 38.16-56.23)	59.24±4.08 (95%CI 50.92-67.57)	0.047

	**Sjogren's Syndrome - ILD**								
	**Baseline**			**1 year follow-up**			**2 years follow-up**		
	**Anti-Ro52kD+ (n = 21)**	**Anti-Ro52kD- (n = 13)**	***p*-value**	**Anti-Ro52kD+ (n = 21)**	**Anti-Ro52kD- (n = 13)**	***p*-value**	**Anti-Ro52kD+ (n = 21)**	**Anti-Ro52kD- (n = 13)**	***p*-value**
**FVC (L)**	2.36±0.16 (95%CI 2.02-2.69)	2.16±0.17 (95%CI 1.77-2.55)	0.407	2.39±0.16 (95%CI 2.05-2.74)	1.88±0.18 (95%CI 1.49-2.28)	0.044	2.28±0.16 (95%CI 1.94-2.62)	2.07±0.17 (95%CI 1.68-2.46)	0.385
**FVC pp (%)**	94.11±4.76 (95CI% 84.23-103.99)	86.15±6.39 (95CI% 72.22-100.09)	0.328	95.81±4.83 (95CI% 85.81-105.82)	75.82±6.58 (95CI% 61.61-90.03)	0.021	91.87±4.83 (95CI% 81.87-101.87)	83.99±6.39 (95CI% 70.11-97.87)	0.334
**DLCO pp (%)**	69.46±4.67 (95%CI 59.87-79.05)	65.13±5.73 (95%CI 52.42-77.85.)	0.564	71.81±4.68 (95%CI 62.33-81.28)	58.33±5.96 (95%CI 45.27-71.39)	0.086	70.82±4.69 (95%CI 61.22-80.42)	57.58±5.73 (95%CI 44.86-70.29)	0.086

Abbreviations: SjS-ILD: Sjögren's syndrome associated interstitial lung disease; ASyS-ILD: anti-synthetase syndrome–associated interstitial lung disease; FVC: forced vital capacity; FVC pp: percent-predicted forced vital capacity; DLCO: diffusing capacity of the lung for carbon monoxide; DLCO pp: percent predicted diffusing lung capacity for carbon monoxide; CI = confidence interval.

Table S7. Comparison of lung function in Anti-synthetase-ILD positive/negative for Ro52kD according to anti-Jo1 or other RSA at different timepoints.

	**ASyS-ILD anti-Ro52kD positive (n = 9)**								
	**Baseline**			**1 year follow-up**			**2 years follow-up**		
	**Anti-Jo1 positive (n = 5)**	**Non-Jo1 ASA+ (n = 4)**	***p*-value**	**Anti-Jo1 positive (n = 5)**	**Non-Jo1 ASA+ (n = 4)**	***p*-value**	**Anti-Jo1 positive (n = 5)**	**Non-Jo1 ASA+ (n = 4)**	***p*-value**
**DLCO pp (%)**	56.38±8.48 (95%CI 55.06-73.09)	48.67±9.20 (95%CI 29.91-67.43)	0.54	51.75±7.97 (95%CI 35.50-67.99)	38.00±9.21 (95%CI 19.24-56.76)	0.263	48.79±8.48 (95%CI 31.62-65.96)	44.81±10.06 (95%CI 24.49-65.12)	0.763
	**ASyS-ILD Anti-Ro52kD negative (n = 24)**								
	**Baseline**			**1 year follow-up**			**2 years follow-up**		
	**Anti-Jo1 positive (n = 10)**	**Non-Jo1 ASA+ (n = 14)**	***p*-value**	**Anti-Jo1 positive (n = 10)**	**Non-Jo1 ASA+ (n = 14)**	***p*-value**	**Anti-Jo1 positive (n = 10)**	**Non-Jo1 ASA+ (n = 14)**	***p*-value**
**DLCO pp (%)**	72.77±5.79 (95%CI 60.99-84.54)	64.08±4.42 (95%CI 59.17-74.99)	0.238	68.73±6.35 (95%CI 55.93-81.53)	67.96±4.70 (95%CI 58.43-77.48)	0.922	58.58±6.37 (95%CI 45.79-71.39)	59.00±4.73 (95%CI 49.48-68.52)	0.958
	**ASyS-ILD anti-Jo1 positive (n = 15)**								
	**Baseline**			**1 year follow-up**			**2 years follow-up**		
	**Anti-Ro52kD+ (n = 5)**	**Anti-Ro52kD- (n = 10)**	***p*-value**	**Anti-Ro52kD+ (n = 5)**	**Anti-Ro52kD- (n = 10)**	***p*-value**	**Anti-Ro52kD+ (n = 5)**	**Anti-Ro52kD- (n = 10)**	***p*-value**
**DLCO pp (%)**	56.38±8.48. (95%CI 55.06-73.09)	72.77±5.79 (95%CI 60.99-84.54)	0.115	51.75±7.97 (95%CI 35.50-67.99)	68.73±6.35 (95%CI 55.93-81.53)	0.1	48.79±8.48 (95%CI 31.62-65.96)	58.58±6.37 (95%CI 45.79-71.39)	0.358
	**ASyS-ILD non-Jo1 ASA+ (n = 18)**								
	**Baseline**			**1 year follow-up**			**2 years follow-up**		
	**Anti-Ro52kD+ (n = 4)**	**Anti-Ro52kD- (n = 14)**	***p*-value**	**Anti-Ro52kD+ (n = 4)**	**Anti-Ro52kD- (n = 14)**	***p*-value**	**Anti-Ro52kD+ (n = 4)**	**Anti-Ro52kD+ (n = 14)**	***p*-value**
**DLCO pp (%)**	48.67±9.20 (95%CI 29.91-67.43)	64.08±4.42 (95%CI 59.17-74.99)	0.138	38.00±9.21 (95%CI 19.24-56.76)	67.96±4.70 (95%CI 58.43-77.48)	0.0057	44.81±10.06 (95%CI 24.49-65.12)	59.00±4.73 (95%CI 49.48-68.52)	0.206

Abbreviations: ASA: Antisynthetase Antibody; ASyS-ILD: anti-synthetase syndrome–associated interstitial lung disease; DLCO pp: percent-predicted diffusing capacity of the lung for carbon monoxide; ARS: aminoacyl tRNA synthetase, CI = confidence interval.

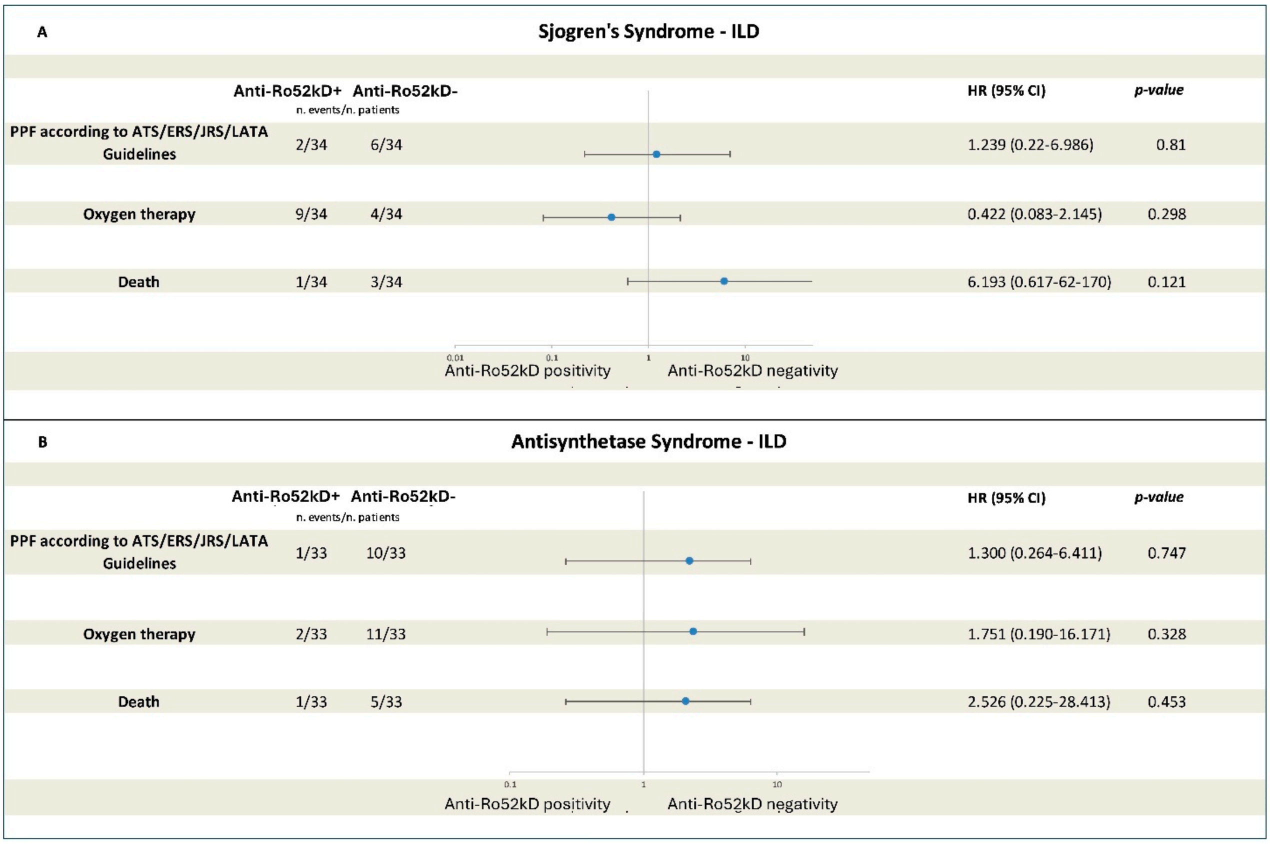

Figure S1. Forest plot of hazard ratios (HRs) adjusted for age and sex comparing anti-Ro52kD-positive versus anti-Ro52kD-negative patients for three time-to-event outcomes in (A) Sjogren's Syndrome-ILD and (B) Antisynthetase Syndrome-ILD: progression to PPF (defined according to ATS/ERS/JRS/ALAT criteria), initiation of oxygen therapy, and all-cause death. Event counts (events/total) for Ro52kD positive and Ro52kD negative groups are shown beside each outcome. *Abbreviations:* ILD: interstitial Lung Disease; HR, hazard ratio; CI, confidence interval; PPF, progressive pulmonary fibrosis; ATS, American Thoracic Society; ERS, European Respiratory Society; JRS, Japanese Respiratory Society; ALAT, Asociación Latinoamericana de Tórax.
